# Magnetically Assisted Control of Stem Cells Applied in 2D, 3D and In Situ Models of Cell Migration

**DOI:** 10.3390/molecules24081563

**Published:** 2019-04-19

**Authors:** Richard Harrison, Jeni Luckett, Sarah Marsh, Hilda Anaid Lugo Leija, Shelanah Salih, Reem Alkharji, Virginie Sottile

**Affiliations:** 1 Wolfson STEM Centre, School of Medicine, The University of Nottingham, Nottingham NG7 2RD, UK; richard.harrison@nottingham.ac.uk (R.H.); stxsamar@exmail.nottingham.ac.uk (S.M.); mzxhl@exmail.nottingham.ac.uk (H.A.L.L.); shelanah.salih@charmouniversity.org (S.S.); rrk-2010@hotmail.com (R.A.); 2 Preclinical imaging unit, School of Medicine, The University of Nottingham, Nottingham NG7 2RD, UK; jeni.luckett@nottingham.ac.uk; 3 College of Medical and Applied Sciences, Department of Medical Laboratory Sciences, Charmo University, Chamchamal 46023, Iraq

**Keywords:** magnetic particle, stem cell, cell therapy

## Abstract

The success of cell therapy approaches is greatly dependent on the ability to precisely deliver and monitor transplanted stem cell grafts at treated sites. Iron oxide particles, traditionally used in vivo for magnetic resonance imaging (MRI), have been shown to also represent a safe and efficient in vitro labelling agent for mesenchymal stem cells (MSCs). Here, stem cells were labelled with magnetic particles, and their resulting response to magnetic forces was studied using 2D and 3D models. Labelled cells exhibited magnetic responsiveness, which promoted localised retention and patterned cell seeding when exposed to magnet arrangements in vitro. Directed migration was observed in 2D culture when adherent cells were exposed to a magnetic field, and also when cells were seeded into a 3D gel. Finally, a model of cell injection into the rodent leg was used to test the enhanced localised retention of labelled stem cells when applying magnetic forces, using whole body imaging to confirm the potential use of magnetic particles in strategies seeking to better control cell distribution for in vivo cell delivery.

Academic Editors: Jon Dobson and Iwona Cicha

## 1. Introduction

Regenerative medicine aims to restore tissue function using the potential of stem cells to regenerate or replace damaged tissues. The successful use of mesenchymal progenitors in a range of preclinical and clinical models in particular support their therapeutic value as cellular and acellular tools for regenerative medicine applications [1]. As a growing range of approaches becomes available to manipulate stem cells in vitro, the development of targeted cell delivery methods is increasingly important to ensure the efficiency of these regenerative approaches in vivo [2]. One such technology recently applied to stem cell research is the use of magnetic micro- and nano-particles referred to as superparamagnetic iron oxide nanoparticles (SPION) and microparticles of iron oxide (MPIO), respectively, which can be made from an iron oxide core surrounded by a protective shell [3].

Iron oxide nanoparticles have been developed as contrast agents employed for advanced MRI imaging in vivo [4,5,6], and more recently as theranostics mediators, enabling the application of targeted hyperthermia for tumour treatment [7]. Their cytocompatibility and capacity for efficient cellular uptake also made them applicable to in vitro studies, facilitated by the availability of commercial fluorescently tagged particles compatible with confocal and advanced super-resolution imaging [8,9,10]. Efficient spontaneous uptake of MPIO has been documented in a range of cell types including human stem cells, and was recently shown to be compatible with nanoparticle-based intracellular sensing [10]. Beyond their use as contrast agent, internalised iron oxide particles can also confer magneto-responsiveness to cells, thereby providing a means to remotely affect cell behaviour through exposure to magnetic force [11].

In the present study, the potential use of MPIO to enhance the spatial control of stem cells through the application of magnetic forces was assessed in a range of experimental models analysing cell responsiveness in vitro and in situ.

## 2. Results

### 2.1. Magnetically Assisted Preferential Seeding

**Figure 1:**
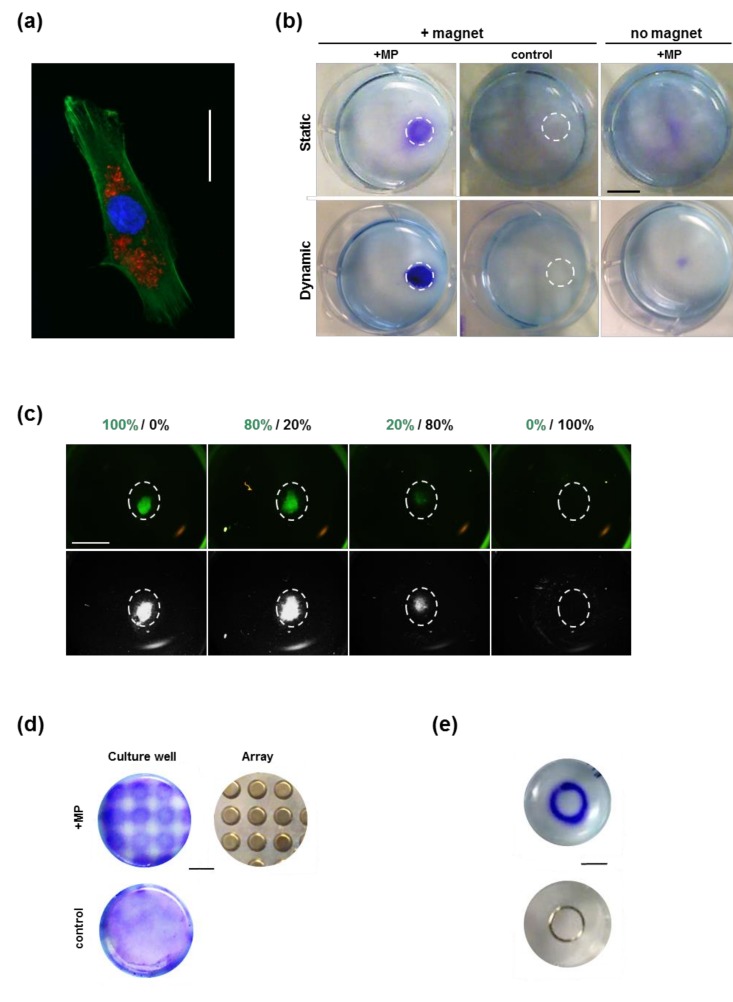
In vitro patterning of MSC seeding using MPs. (**a**) Fluorescence imaging of phalloidin-stained MSCs (green) showing internalised MPs (red) with dapi nuclear counterstain (blue). Bar = 50 μm. (**b**) Toluidine blue staining 24 h after MP-labelled or unlabelled MSCs were seeded in static (top panel) or dynamic (bottom panel) conditions in the presence or absence of a magnet located under the culture well (white dashed circle). Bar = 10 mm. (**c**) Fluorescence imaging of the adherent population resulting from the incubation of MP-loaded GFP-positive (green) and unloaded GFP-negative MSCs mixed at different ratios (indicated as respective percentages), in the presence of a magnet placed under the plate (white dashed circle) for 48 h. Top panel: fluorescence channel, bottom panel: visible channel. Bar = 5 mm (**d**) Toluidine blue staining (left panel) 24 h after wells placed on a magnet array (right) were seeded with MP-labelled (+MP) or unlabelled (control) MSCs. Bar = 9 mm. (**e**) Patterned cell distribution (top left) observed 48 h after seeding MP-labelled MSCs in a well placed on a metallic ring (top right) in contact with a magnet, resulting in matching cell seeding as seen after toluidine blue staining (blue). Bar = 10 mm. See image analysis in Figure S1.

Incubation of mesenchymal stem cells (MSCs) with fluorescently tagged magnetic particles (MP) for 24 h was used to efficiently label cells intracellularly (Figure 1a). The capacity of this intracellular labelling to confer magnetic responsiveness and targeted cell seeding was evaluated in vitro by comparing the seeding arrangement in the presence or absence of a magnet located under the culture well (Figure 1b). The presence of a magnet resulted in the patterned seeding of MP-labelled cells, which was not seen in the absence of magnet, or when unlabelled cells were exposed to a magnet. Similar patterned seeding was achieved both in static conditions, when the cell suspension was added to a stationary plate, and under dynamic seeding conditions, when the plate was subjected to orbital shaking during the incubation time.

The specificity of this MP-driven cell response was tested in a competition experiment, where MP-containing green fluorescent MSCs were mixed with increasing numbers of control unlabelled MSCs, and exposed to the apposition of a magnet under the well (Figure 1c). Observation of the resulting cell pattern showed concentration of the fluorescent signal overlapping with the magnet location, with a level of intensity proportional to the ratio of MP-loaded cells.

The magnetic responsiveness of MP-loaded MSCs was further tested using a 96-well format (Figure 1d) and a ring (Figure 1e) magnetic array, which both produced precisely matched 2D seeding patterns after 1 and 2 days in culture, respectively.

### 2.2. Models of Cell Recruitment

**Figure 2:**
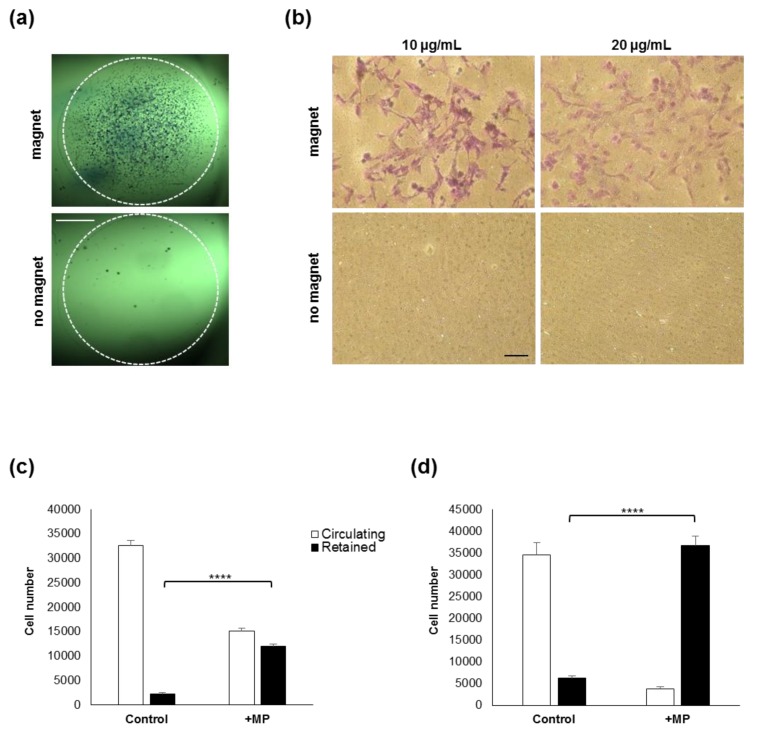
Magnetically assisted recruitment of MSCs in vitro. (**a**) Representative images of toluidine blue staining of labelled MSCs recruited anti-gravitationally from hanging drops following 48 h incubation in the presence (top) or absence (bottom) of a magnet. Dashed circle shows the edge of the drop. Bar = 1 mm. (**b**) In vitro recruitment of adherent cells, labelled with the standard (10 µg/mL) and double (20 µg/mL) concentration of MP, attached onto the underside of an insert membrane upon 48 h magnet exposure, visualised by toluidine blue staining. Bar = 100 μm. (**c**,**d**) Retention of circulating MSCs in vitro, whereby unlabelled (control) or MP-labelled (+MP) cells flowing in a circulation model were exposed to the presence of a small (**c**) or large (**d**) magnet for 30 min, before the proportion of cells either immobilised (black bars) or remaining in circulation (white bars) was quantified (**** *p* < 0.0001).

To further evaluate the ability of the internalised MP to promote cell recruitment towards a magnetic source, an anti-gravitational model was established, as MP-loaded MSCs were cultured in hanging drops incubated beneath a magnet for 48 h. Labelled cells showed upwards migration and subsequent attachment to the upper surface, while unlabelled cells remained unattached (Figure 2a). When adherent MP-loaded cells were incubated for 48 h in the presence of a magnet placed in an in-well insert positioned above the well (Figure 2b), cells attached to the underside of the insert were fixed and visualised following toluidine blue staining, which showed MP-loaded cells had been recruited, while no cells could be seen with unloaded controls.

### 2.3. Cell retention in a Model of Circulating Cells

Following the magnetic recruitment of cells in 2D culture models, a flow-through system was set up to test the magnetic retention of circulating MSCs using magnets applied to the side of the tubing (Figure 2c,d). While unlabelled cells flown through as control remained in the circulating fraction, a significant proportion of MP-labelled cells was immobilised and retained at the side of magnet apposition (Figure 2c). The retained fraction increased when a stronger magnet was used, leading to near complete entrapment of flowing cells at the magnet site (Figure 2d).

### 2.4. Directed Migration of Adherent Cells

**Figure 3:**
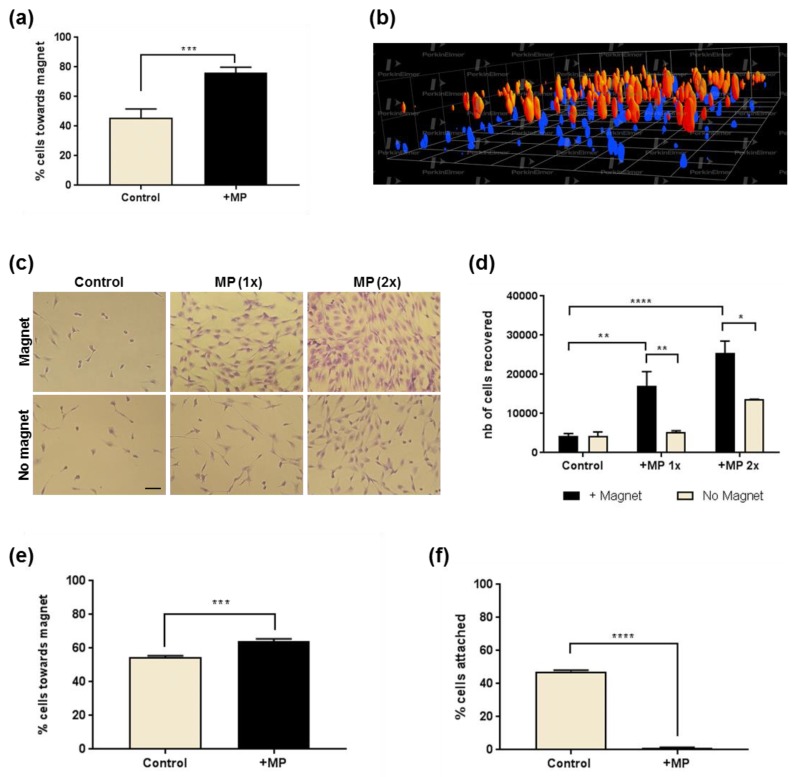
Magnetically assisted MSC migration in culture environments. (**a**) 2D cell migration of unloaded or MP-loaded cell populations exposed to a magnet located under the culture plate laterally to the field of view, presented as the proportion of cells showing net cell movement towards the magnet side. (**b**) Confocal imaging of cell distribution of labelled (blue) or unlabelled (red) cells seeded on a 200 μm porous membrane and exposed to magnet presence for 72 h. (**c**) Migration of MSCs in a 3D hydrogel in the presence of MPs. Toluidine blue staining of adherent MSCs recruited at the bottom of the plate after migration through a gel after 24 h in the presence or absence of a magnet located underneath the well. Bar = 100 μm. (**d**) Corresponding metabolic activity measurement of adherent MSCs loaded with 10 µg/mL (1×) or 20 µg/mL (2×) MP dose and recovered after migration through a gel in the presence (white) or absence (black) of a magnet positioned underneath the well. (**e**,**f**) Effect of a magnet located on the side of the well containing MSCs seeded in a gel, with or without MP loading, showing the percentage of cells displaying a move towards the magnet side (**e**), and the percentage of cells reaching the base of the well (**f**). * *p* < 0.05, ** *p* < 0.01, *** *p* < 0.001 and **** *p* < 0.0001.

To better evaluate the magnet-assisted migratory response conferred by internalised MP, adherent MP-loaded MSCs were incubated in the presence of a magnet placed lateral to the field of view, and their spatial distribution was analysed. Analysis of cell migration by time-lapse microscopy showed significantly more MP-loaded cells showing net directionality towards the magnet compared to controls incubated in the absence of magnet (Figure 3a).

### 2.5. 3D Cell Recruitment

Next, experiments were designed to evaluate the magnetic recruitment for cells seeded in 3D environments (Figure 3b–f). In the first model, cells seeded onto a porous membrane were exposed to a magnet for 72 h before imaging to analyse their distribution (Figure 3b). Confocal imaging revealed MP-loaded cells were found closer to the apposed magnet than control cells. In a second model, control and MP-loaded cells seeded in a hydrogel were incubated above a magnet array for 72 h, and cells which had migrated vertically through the gel and reached the bottom of the plate were imaged (Figure 3c) and semi-quantified (Figure 3d) using a metabolic assay. Results obtained highlighted a significant migratory response of the MP-loaded cells exposed to the magnetic field when compared to no magnet or no MP controls. An opposite approach taken to evaluate the retention of cells seeded in a gel confirmed the significant response of MP-loaded MSCs exposed to a magnet (Figure 3e,f).

### 2.6. In Situ Cell Retention in Injection Models

**Figure 4:**
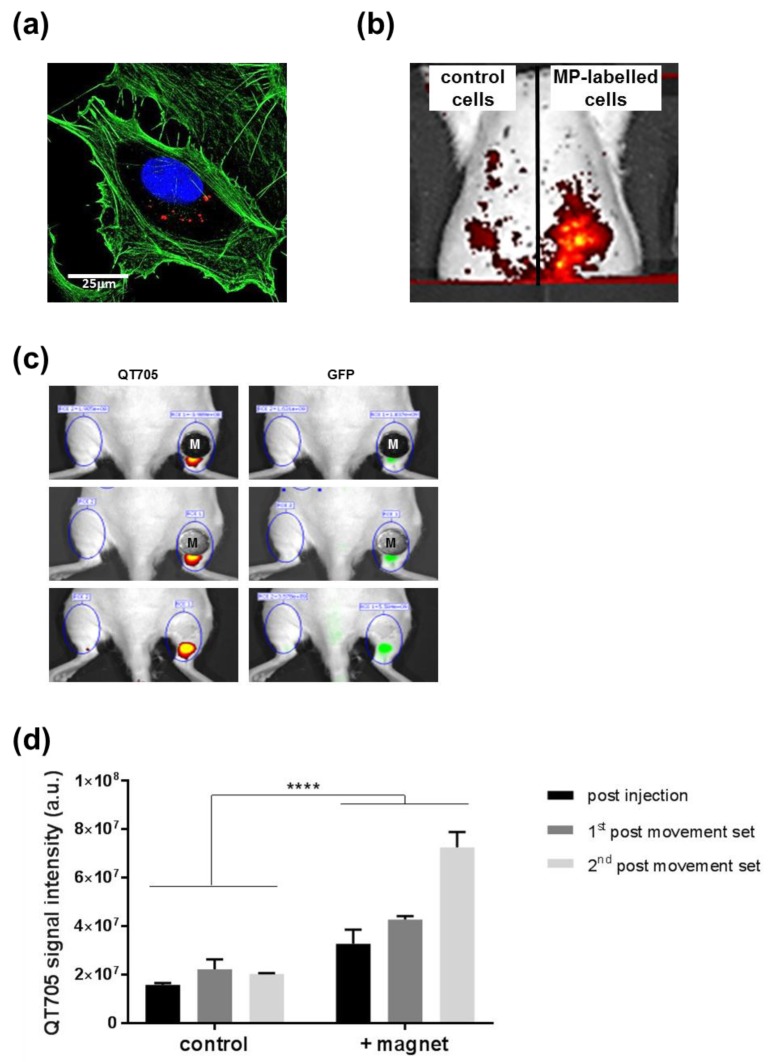
Whole body imaging of MP-labelled cells injected into a rat model. (**a**) QT705 (red) used for fluorescence labelling of MSCs, shown with Phalloidin staining (green) and dapi (blue) counterstain. Bar = 25 μm. (**b**) Peritoneal injection of MP-labelled (right) or unlabelled (left) MSCs in the presence of subcutaneously implanted magnets, imaged using the IVIS system. (**c**) MP-labelled cell suspension marked with QT705 assessed for fluorescence detection after subcutaneous injection in the hind limbs in the presence of dermally applied magnets. Imaging was carried out immediately after injection (top panel), after a first set of 20 bilateral leg flexion movements (middle panel), and after a second set of flexion movements post magnet removal (bottom panel). Bilateral comparison between cell fluorescence upon subcutaneous injection with (right) or without (left) magnet application. (**d**) Relative increase in QT705 fluorescence signal observed in the presence (+magnet) or absence (control) of a magnet, measured immediately after injection (post injection) or after 1 set (1st post-movement set) or 2 sets (2nd post-movement set) of flexion movements. **** *p* < 0.0001.

To test whether the magnetically assisted 3D cell retention observed in vitro could lead to cell delivery applications, an injection model in rat tissue was set up using quantum dots (QT705) to label MSCs (Figure 4a) for whole body imaging. In the presence of a rod magnet implanted intramuscularly, subcutaneous delivery of control or MP-loaded cells was performed (Figure 4b). Post-injection whole body imaging showed stronger QT705 signal for MP-labelled cells, indicating a higher concentration of cells compared to the low signal observed for control cells. A less invasive approach was then taken to position a magnet on the surface of the skin before subcutaneous injection in the right hind limb, to evaluate the retention of MP-loaded MSCs compared to the contralateral control hind limb injected without magnet (Figure 4c). Imaging after injection showed stronger signal on the magnet side compared to control (top panel), which was maintained after the execution of 20 bilateral leg flexion movements (middle panel). After removal of the magnet, 20 further flexion movements were applied before final imaging (lower panel), which confirmed increased cell retention at the site of injection on the magnet side, as shown by semi-quantitative imaging of the QT705 signal (Figure 4d).

## 3. Discussion

### 3.1. Use of MP-Labelled Cell Populations:

Magnetic particles are widely used for magnetic resonance imaging, and have been used for magnetic cell labelling in this context [12,13,14,15]. They are increasingly involved in intracellular cell labelling strategies for tracking and imaging of a range of therapeutically relevant cell populations in vitro and in vivo [5,16,17,18,19]. In addition to MRI applications exploiting their iron core, functionalised MPIO have also been developed to enable detection in other modalities such as PET and fluorescence microscopy [20,21,22]. SPION and MPIO are increasingly used as therapeutic agents for interventions such as hypothermia targeting cancer cells [23], in advanced drug delivery approaches, and more recently in receptor activation through mechano-sensitive and thermo-responsive targets [24,25,26,27]. Silica-coated iron oxide particles enable efficient cell labelling without the need for adjuvants, and have demonstrated both high cytocompatibility and maintenance of mesenchymal stem cell multipotency [8].

Since particles can be readily functionalised [28,29], the exploitation of their magnetic properties has been envisaged for magnet-driven drug delivery approaches, whereby particles might act as vector for a therapeutic drug or cargo which could be localised through magnet exposure, for applications in joint, spinal cord, ear and eye treatments for instance [30,31,32]. Building on this concept of magnetic targeting, the use of iron oxide particles to label cells intracellularly has also been proposed as a way to confer them magneto-responsiveness [2,3,33]. Iron oxide particle internalisation for cell labelling was shown to be safe in a range of cell types using in vitro and in vivo models [9,34]. This represents a promising solution to develop better cell targeting and improve stem cell localisation and engraftment for tissue engineering. While some encouraging observations have been reported for new cell delivery approaches with magnetic forces, these have remained exploratory and largely based on divergent experimental models. The present study aimed to examine a range of accessible in vitro and ex vivo models which could accelerate the systematic study of magnetically labelled cell responses, and help refine parameters to achieve better spatial control.

### 3.2. Spatial Control of In Vitro Cell Distribution

Results presented here highlight the application of magnetic forces to control MSCs in culture using experimental models of cell recruitment, immobilisation and patterning. The design of magnetic arrays has been proposed as a tissue assembly and culture engineering tool [35,36], which could easily be combined with the labelling approach presented here, exploiting the efficient spontaneously internalisation of silica-shell MPIO by MSCs and other cell types [8,9]. This method may be easier and more readily accessible than other multicell assembling methods such as optical tweezers, which depend on highly advanced technology [37].

The cell accumulation models used here also demonstrate how MP-labelled MSCs could be recruited to a site through non-invasive magnet exposure, as labelled cells could be concentrated whether they were circulating as a suspension, or attached onto a seeded surface. These results support other observations from different injection models carried out in and ex vivo, where the presence of magnets was shown to enhance the concentration of particle-labelled cells at the intervention site. Several preclinical studies have targeted intra-articular injections as a potential application for magnetically assisted cell retention. The difficulty posed by the clearance of treatments delivered to the joint space requires strategies enabling the sufficient retention of injected therapies to the target site [38]. In small animal models, cells delivered into the joint space have shown concentration and accumulation when an external magnet was present. This was demonstrated when cell distribution was analysed in tissues immediately after injection [39], as well as in post-injection follow-up in vivo [40,41,42]. In line with results observed here in a subcutaneous injection model, the initial cell retention from temporary magnet application during the injection appeared to be maintained even after subsequent movement and mechanical challenge. In the orthopaedic field, too, a rat bone fracture model was used to test a similar approach [43], whereby iron oxide particle-labelled cells delivered to a non-union bone defect, in the presence of a magnetic source, were enriched at the intervention site and correlated with better healing scores. In a cardiac injection model, magnets applied during intra-myocardial infusion of particle-labelled cardioprogenitors were also associated with higher cell retention and better myocardial performance [44,45]. Additional preclinical models shown in the literature to benefit from localised magnet-assisted cell delivery include intravitreal ocular injection [46], and neurological applications using iron oxide particles in vitro and in vivo [31,47,48,49].

In the more challenging case of systemic cell administration, recent reports have described a magnet-driven approach for the trapping of stem cells after systemic injection for wound healing. When tested in vivo in mouse [50], a magnet was implanted subcutaneously for the duration of the injection of particle-labelled cells into the circulation. This resulted in an immediate increase in cells detected near the magnet site, which was sustained after 24 h. A comparable study applying daily magnetic exposure also observed enrichment at the wound site [51].

These promising results suggest that particular cell therapy approaches may benefit from magnet-based enrichment; however careful optimisation of the magnet (strength, shape, placement) and of particle loading parameters, using techniques including magnetophoresis [33], is required to define the precise operating procedure specific for each target tissue. A limitation of the present study was the use of a post-mortem tissue injection model, which although common in the field [39] and sufficient to provide proof of concept would need further validation. In vivo follow-up testing is needed, in particular, to evaluate both insufficient retention leading to undesirable cell migration away from the intended site and detrimental cell over-aggregation from excessive strength leading to poor engraftment.

### 3.3. Magnetically Assisted Stem Cell Recruitment

In addition to the accumulation of cells present in physiological fluid, magnetic forces may also be a useful tool to recruit anchored cell populations. Magnet exposure was here able to recover adherent cells, suggesting possible applications for in vitro processes such as cell passaging step for scale-up culture, which could benefit from alternative options to standard enzymatic methods typically used for adherent cell harvest [52]. It is unclear, however, to what extent such cell recovery from attached cultures might be facilitated by the loss of attachment occurring during cell division, which could introduce a bias in the subpopulation recovered.

In the case of adherent cells recruited towards a magnetic source, there is evidence supporting the present observations that cell migration properties can be enhanced [53], and that adherent cells can be directed towards the magnetic source [11]. Changes in cell orientation and morphology have been described when exposing cells to magnetic arrays in vitro [54]. However, there is little information available to estimate and model the strengths required to force the movement of adherent cells in a set direction, and this is likely due to the complexity of integrating the different intrinsic parameters such as the nature of cell-substrate attachment, the resistance of cell membranes to the pull from the internalised iron oxide particles, and the kinetics of cytoskeleton rearrangements, which are likely to show some level of cell type-specificity. Magnet-driven migration of adherent cells was also achieved through a 3D gel structure and through a porous membrane; however, careful controls are essential with these models to precisely account for the effect of gravity when assessing downwards vertical movement. For cell recruitment models, it also remains unclear how long the phase of magnet exposure should be after injection to confer an optimal advantage in terms of cell enrichment in vivo: while several studies applied the magnet for as little as 10 min [40,41,42,43], others used it for 4 h [39], or even in some cases daily 6 h repeats [51]. Similarly, the reported particle doses used for cell loading could vary, supporting the need for a more systematic approach to defining the optimal parameters adapted to each clinical model.

### 3.4. Perspectives for Therapeutic Applications

The use of fluorescently labelled MPs has advantages beyond their use for whole body imaging: they are amenable to fluorescent, confocal and super-resolution microscopy [10]. Such particles could be further developed as therapeutic agents, acting both as cell anchors and as cell activators, as their coating or formulation could be functionalised to also provide a biological benefit to host cells [55]. In terms of safety, the external magnets employed here (0.3T–0.4T) and in other studies (0.1T–1.3T) [3,37,38,40,41] investigating magnet-assisted delivery are within the range of widespread MRI systems (0.5T), and below typical clinical MRI equipment (1.5T–3T) [56], suggesting clinical safety. Although rigorous validation is required, the magnet strengths considered for such assisted delivery applications are thus modest compared to those approved for patient use, and to 9T preclinical systems.

Future therapeutic applications of these magnet-based strategies could significantly enhance the efficiency of cell therapies, by increasing the actual cell dose achieved at the target site and maximising engraftment while minimising adverse side effects [2,46]. One key consideration is the rapid distance-dependent drop in magnetic pull away from the surface of the magnet [2], which may restrict such approaches to intervention sites close to where magnetic sources can be safely applied or implanted [3,45]. These preclinical observations are also supportive of further advances in medical engineering towards the development of magnetised surgical implants to promote cell recruitment, as suggested in a recent publication proposing the use of magnetised stents to enhance cell retention for cardiovascular treatment [57]. Such an approach has recently shown promising results in a rat model of stent angioplasty [58], whereby endothelial cells were successfully recruited to the carotid artery using magnetic exposure, and showed a beneficial effect through reduced stenosis after 2 months.

## 4. Materials and Methods

Reagents were purchased from Thermo Fisher Scientific (Loughborough, UK), and Neodymium magnets were purchased from Magnet Expert, UK (http://www.first4magnets.com/) unless otherwise stated. The magnet layouts used in vitro are presented in Figure S2.

### 4.1. Cell Culture and Labelling

A human bone marrow mesenchymal stem cell line (MSCs) [8,59] was cultured and expanded under standard cell culturing conditions (37.5 °C, 5% CO_2_) in standard culture medium consisting of DMEM supplemented with 10% (*v*/*v*) FBS, 1% (*v*/*v*) non-essential amino acids, 1 mM L-Glutamine, 1 mM Pyruvate and 1% Penicillin/streptomycin. Cells were passaged using Trypsin/EDTA.

MSC labelling was performed with 1000 nm fluorescent magnetic particles (MP, Chemicell, Berlin, Germany), as previously described [8]. In brief, adherent cell populations were incubated with MPs (standard dose 10 μg Fe/mL) added to the culture medium for 24 h, before a thorough wash with phosphate buffered saline (PBS) and medium change the next day to remove excess particles. For fluorescent imaging, labelled cells were stained with Alexa Fluor 488 phalloidin and Hoechst 33342 (Sigma Aldrich, Gillingham, UK) as nuclear counterstain as previously described [8].

### 4.2. In Vitro Cell Patterning

Unlabelled and MP-labelled cells were added at 10^5^ cells/mL to well-plates and incubated on top of disc magnets (8 mm × 4 mm, 3600 Gs), in static conditions or with orbital shaking, for 24 h. Cells were then fixed for 15 min with 4% cold paraformaldehyde (PFA, VWR, Lutterworth, UK) prior to staining with toluidine blue (0.1% for 10 min). For the competition experiment, fluorescent microscopy was used to analyse the seeding pattern resulting from the incubation of MP-loaded GFP-positive MSCs [8] mixed with increasing ratios of unloaded GFP-negative MSCs and seeded in a 12-well plate in the presence of disc magnets (8 mm × 4 mm, 3600 Gs) for 48 h. Patterned seeding was analysed using the 96-well format magnetic array of a Magnefect-LT (nanoTherics, Newcastle under Lyme, UK). MP-loaded or unloaded cells were seeded into wells of a 6-well plate placed on the magnetic array for 24 h before PFA-fixation and toluidine blue staining. In a parallel experiment, MP-labelled cells were also added to a 12-well plate placed on a steel metallic ring on top of a disc magnet (12 mm × 4 mm, 3250 Gs) for 48 h. Cells were then fixed, stained with toluidine blue, imaged and analysed using Image J (NIH, Bethesda, MD, USA) to quantify staining intensity.

### 4.3. In Vitro Cell Recruitment

*Circulating cell retention model:* Magnetic entrapment of cells was examined in a perfusion system set up with a PHD 2000 High Force Syringe Pump (Harvard Apparatus, Cambourne, UK) with syringes and 2 mm internal diameter PTFE tubing. Unlabelled and labelled cells were harvested using standard culture methodology and resuspended in 10 mL of complete medium at 5000 cells/mL. Small (8 mm × 4 mm) or large (12 mm × 4 mm) neodymium magnets were placed on the tubing through which cells were perfused at 333 µL/min for 30 min, with a shear stress of 5.09 × 10^−2^ dyn·cm^−2^ and a shear rate of 7.07 s^−1^. Cell samples retained in the tubing and remaining circulating cells were then recovered and quantified with a calibrated Presto blue assay according to the manufacturer’s instructions.

### 4.4. In Vitro Migration Analysis

#### 4.4.1. Gel Migration Models 

For the vertical gel migration assay, MP-labelled MSCs (using 10 μg Fe/mL or 20 μg Fe/mL as stated) were seeded at 10^4^ cells/mL in a 0.25% PepGel hydrogel matrix (PepGel, USA) and after 24 h the plates were incubated in the presence or absence of a 24-well plate magnetic array for a further 72 h. The gels were then removed and adherent cells recruited at the bottom of the cells were fixed before staining with Toluidine blue, imaging using the Operetta High Content Imaging System (Perkin Elmer, Beaconsfield, UK) set to scan all the bottom of the well, and cell counting using the Columbus analysis system (Perkin Elmer, UK).

For the lateral gel migration assay, loaded and unloaded cells were mixed with HistoGel and seeded in a 24-well plate at 10^4^ cells/mL, incubated for 4 h to allow gel setting before exposure to 10 × 5 mm neodymium magnets fixed to the side of the well. After 5 days the magnets were removed, cells were fixed and stained with toluidine blue to allow for imaging and cell counting.

#### 4.4.2. Cell Migration through a Porous Scaffold

5 × 10^5^ cells, labelled or unlabelled, were seeded onto Alvetex scaffolds (Reinnervate, Sedgefield, UK) and allowed to attach for 24 h before placing on neodymium disc magnets for 72 h. Cells were fixed with PFA and stained with Hoechst 33342 (Sigma-Aldrich) prior to imaging using the Operetta High Content Imaging System and Columbus analysis system using the z-stack function, with 21 fields of view imaged for each scaffold.

#### 4.4.3. Suspended MS Recruitment

For the anti-gravitational cell recruitment model, hanging drops were established in quadruplicate using MP-labelled MSCs alongside unlabelled control cells. 20 µL drops of 10^5^ cells/mL were deposited on the underside of a multiwell plate lid and then placed above humidified wells. A neodymium magnet (10mm × 3mm, 2800 gauss) was placed above each well for 24 h, and the cells attached to the under surface of the lid were fixed, stained with Toluidine blue (0.1% for 10 min) and imaged using an EVOS XL Core microscope (Peqlab, Lutterworth, UK).

#### 4.4.4. Adherent MS Recruitment 

MSCs seeded onto microcarriers (Corning, Flintshire, UK) were labelled with MPs at the standard dose (10 μg Fe/mL) or double dose (20 μg Fe/mL) for 24 h. Millicell cell culture inserts (Merck Millipore, Gillingham, UK) holding two magnets (5 mm × 4 mm, 4000 Gs) were then placed in each well, while empty inserts were used as control. After 48 h, inserts were collected and the cells attached to the underside were fixed with PFA, stained with 0.1% Toluidine blue, and imaged using a Nikon Eclipse TS100 inverted microscope (Nikon, Kingston upon Thames, UK).

#### 4.4.5. 2D Cell Migration Analysis 

For time lapse imaging, MP-labelled cells seeded in 6-well plates were incubated in the presence or absence of a magnet (10 mm × 3 mm, 2800 Gs) placed underneath the plate, lateral to the field of view. Live cell imaging was carried out using a JuLi Fl Stage system microscope (NanoEnTek, Seoul, Korea) with images taken every 7 min for up to 8 h. Cell tracks were analysed using the MTracker plugin from Fiji (Madison, WI, USA). 20 cell tracks were collected for each condition.

### 4.5. Whole Body Imaging

#### 4.5.1. Quantum Dot Labelling

Qtracker^®^ 705 (QT705) was used to fluorescently tag MSCs (with or without MP-labelling) for the whole body imaging work, according to the manufacturer’s instructions. A 10^7^ cells/mL suspension was added to the Qtracker component mix and incubated for 60 min at 37 °C, then washed twice with complete medium before use.

#### 4.5.2. Tissue Injection Models

Wistar rats were culled by carbon dioxide asphyxiation immediately prior to the procedures. For the subcutaneous magnet injection model, diametrically magnetised neodymium rod magnets (3 × 12 mm, 3000 Gs) were implanted subcutaneously in the left and right abdominal regions and the wound was sealed using Dermafuse™ cyanoacrylate tissue adhesive (Mo-Sci Corporation, Rolla, MO, USA). MP-labelled cells and unlabelled control cell suspensions (100 µL containing 10^6^ cells) were injected to left and right regions respectively, and left to rest for 20 min before imaging using the IVIS Spectrum In Vivo Imaging System (Perkin Elmer, UK). For the dermally applied injection model, MP-labelled cell suspensions (100 µL containing 10^6^ cells) were injected to each hind limb, in the presence (right) or the absence (left) of a neodymium magnet (12 mm × 4 mm, 3250 Gs) applied to the joint region and held in place using GLUture (Abbott Laboratories, Maidenhead, UK) respectively. After transfer to the IVIS Spectrum imaging stage, the area was imaged immediately to produce the initial images. 20 full flexion movements were applied to each hind limb, and the region was re-imaged twice (once directly after movement and once after removal of the magnet). Images were recorded at 605/660 and 705 regions, and then adaptive fluorescent background reduction was applied to produce the composite. Experiments were repeated on 5 animals.

### 4.6. Statistical Analysis

Statistical analysis was in the form of ANOVA performed using PRISM (GraphPad Software, version 7, San Diego, CA, USA). Tukey’s post hoc analysis was performed to determine significance between subgroups. Significance shown as * *p* < 0.05, ** *p* < 0.01, *** *p* < 0.001 and **** *p* < 0.0001.

## Supplementary Materials

The following are available online at https://www.mdpi.com/1420-3049/24/8/1563/s1, Figure S1: Image analysis for Figure 1e showing toluidine blue signal intensity across the well midline and the geometry of the ring structure used (red). (n = 3 measurements). Figure S2: Graphical representation of the in vitro layouts used, showing the magnet (M) position and direction of movement (grey arrow).
