# Supplementary material for: Magnetically Assisted Control of Stem Cells Applied in 2D, 3D and In Situ Models of Cell Migration
Source: Molecules. 2019 Apr 19;24(8):1563. doi: 10.3390/molecules24081563 (PMC6515403; doi:10.3390/molecules24081563)
Supplement: Supplementary file 1 [file molecules-24-01563-s001.pdf]

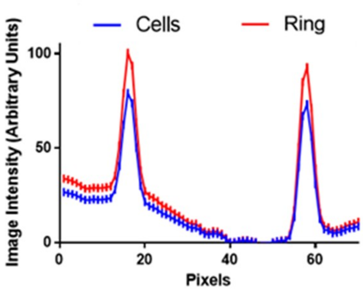

Figure S1. Image analysis for Figure 1e showing toluidine blue signal intensity across the well midline and the geometry of the ring structure used (red). (n = 3 measurements).

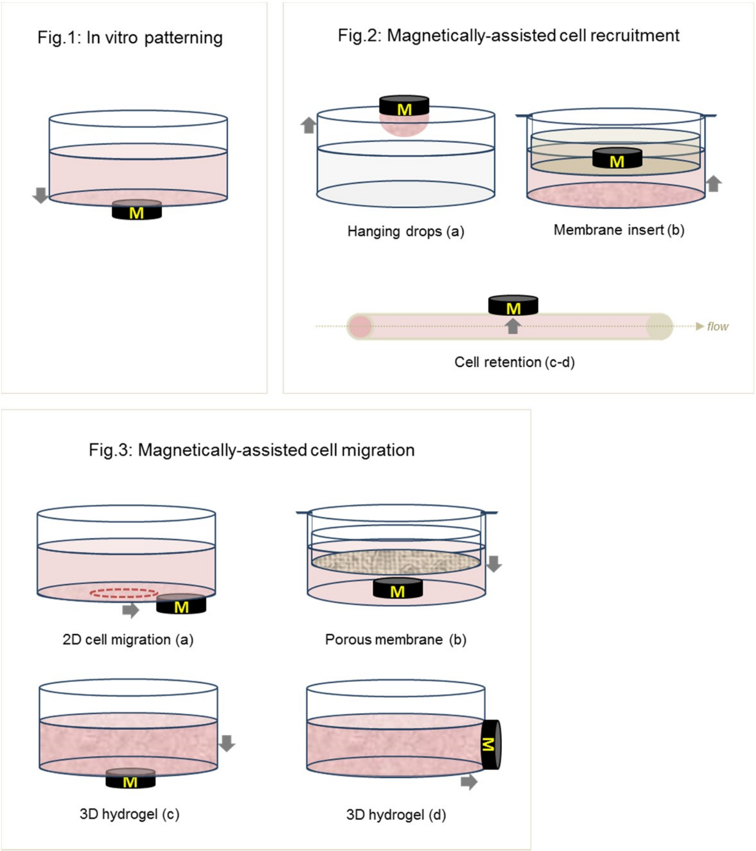

Figure S2. Graphical representation of the in vitro layouts used, showing the magnet (M) position and direction of movement (grey arrow).
